# Physician behaviours that optimize patient‐centred care: Focus groups with migrant women

**DOI:** 10.1111/hex.13110

**Published:** 2020-07-24

**Authors:** Anna R. Gagliardi, Claire Kim, Bismah Jameel

**Affiliations:** ^1^ Toronto General Hospital Research Institute University Health Network Toronto Ontario Canada

**Keywords:** emigrants and immigrants, focus groups, health‐care disparities, patient‐centred care, quality of health care, refugees, women's health, women's health services

## Abstract

**Background:**

No prior research studied how to implement patient‐centred care (PCC) for migrant women, who face inequities in health‐care quality. This study explored migrant women's views about what constitutes PCC and how to achieve it.

**Design:**

We conducted a qualitative study involving three focus groups with migrant women living in Toronto, Canada, recruited from English language classes at a community settlement agency, used constant comparative technique to inductively analyse transcripts and interpreted themes against a published PCC framework.

**Participants:**

Twenty‐three migrant women aged 25‐78 from 10 countries participated.

**Results:**

Women articulated 28 physician behaviours important to them across six PCC domains: foster a healing relationship, exchange information, address concerns, manage uncertainty, share decisions and enable self‐care. They emphasized the PCC domain of exchanging information, which included 13 (46.4%) of 28 behaviours: listen to reason for visit, ask questions, provided detailed explanations, communicate clearly, ensure privacy and provide additional information. Women said that instead of practising these behaviours, physicians rushed through discussions, and ignored or dismissed their concerns and questions. As a result, women said that physicians may not fully understand their problem, and they may refrain from stating important details or avoid seeking care.

**Conclusions:**

This research characterized the lack of PCC experienced by migrant women and revealed specific physician behaviours to optimize PCC for migrant women. Research is needed to develop and evaluate the impact of strategies targeted at migrant women, physicians and health‐care systems to support PCC for migrant women.

## 1 BACKGROUND

According to the World Health Organization, the total number of international migrants (immigrants and refugees) rose 49% over the last decade from 173 million to 258 million, a considerable movement of humans from countries of origin to new countries.^1^ Migrants are less likely than the general population to experience high‐quality health care due to a variety of patient, clinician and system‐level factors.^2^ For example, a systematic review (67 studies, 1996‐2009) of population‐based studies involving American immigrants found they were less likely to have medical insurance, access to a regular health‐care provider, or receive preventive care, tests or services; and more likely to report not being engaged by clinicians.^3^ A scoping review (27 studies, 1993‐2014) found that access to and quality of primary care was influenced by migrant culture (ie social stigma of disease), communication (ie language skills) and socioeconomic (ie unable to attend appointments due to multiple jobs or shift work) factors.^4^ An integrative literature review (35 studies, 1997‐2015) revealed that clinician cultural competence, including skills, awareness, knowledge and personal characteristics influenced communication with migrants.^5^ Hence, strategies are needed to improve migrant health‐care experiences.

The World Health Organization, in collaboration with the United Nations and the International Organization for Migration, generated the Global Action Plan, a framework of priorities to promote the health of migrants.^1^ The Plan emphasizes the need to improve the quality, acceptability, availability and accessibility of health‐care services for all migrants, and in particular, advocates for improving the health and well‐being of women given considerable evidence of persistent gendered inequities in health‐care quality in both developed and developing countries. Patient‐centred care (PCC) is one approach to reduce gendered inequities in health‐care quality that was emphasized in 1995 by Women of the United Nations, in 2009 by the World Health Organization, and in 2018 by the United Nations report Gender Equality in the 2030 Agenda for Sustainable Development.^6^, ^7^, ^8^ PCC is a multidimensional approach to care that is proven to enhance a range of patient‐important and clinical outcomes.^9^ PCC refers to partnership with patients and care partners to tailor care to clinical needs, life circumstances and personal preferences, and equip them with skills, knowledge, and awareness of services to optimize self‐care and quality of life.^9^ The intent is that applying actions across the multiple dimensions of PCC optimizes the care experience and tailors care by taking into account the unique characteristics and skills of individuals. Thus, PCC could improve migrant health‐care experiences, particular for women who face inequitable quality of care.

A scoping review (83 studies, 1990‐2015) of interventions used to improve the health of migrants revealed that most were prevention strategies for diabetes and cardiovascular disease.^10^ A systematic meta‐review (28 reviews, 2011‐2017) identified informational and educational interventions for patients or clinicians that can support PCC.^11^ However, neither review defined PCC or employed a PCC model by which to describe or assess PCC, or identified interventions that support PCC for women. A clinical guideline on preventive health care for migrants addressed women’s health by noting the need to provide culturally sensitive, patient‐centred counselling for contraception, human papillomavirus vaccination and screening of cervical abnormalities.^12^ However, the guideline, based on research published from 1996 to 2010, is not current; lacks specific behaviours for delivering PCC to migrant women; and narrowly focuses on reproductive health, when women's health has evolved to include health issues across the lifespan.^13^ There is limited guidance on how to deliver PCC for migrants.

Another scoping review to identify determinants of PCC specifically for migrant women revealed a paucity of research on this topic (16 studies, 2010‐2019).^14^ Among the five studies that explored migrant women's views and experiences, women said that clinicians ignored or dismissed their concerns, provided little information about potential or actual complications, and behaved in a manner they considered disrespectful. In remaining studies, clinicians noted a lack of training or guidelines to help them care for migrants. No studies evaluated strategies or interventions targeted at either migrants or clinicians to improve PCC. Insight is lacking on how to implement PCC for migrant women across the lifespan. The aim of this study was to explore the views of migrant women about what constitutes PCC and how to achieve it.

## 2 METHODS

### 2.1 Approach

Given few prior studies on PCC for migrant women, we used a basic qualitative descriptive research design to thoroughly explore women's PCC views and recommendations.^15^ This approach does not employ or generate theory; instead, it elicits insight from participants about health services and how services can be improved based on their lived experience. We conducted in‐person focus groups rather than individual interviews because group discussion encourages synergistic conversation about novel or complex issues.^16^ This study was approved by the University Health Network Research Ethics Board. We complied with the Consolidated Criteria for Reporting Qualitative Research.^17^

### 2.2 Sampling and recruitment

We used convenience sampling to recruit migrant women aged 18 + from a community settlement agency that assists newcomers to <city name>, <country's> largest city, which features a diverse multicultural population. The agency serves 20 000 immigrants and refugees annually by offering programs that support family development, healthy lifestyles and employment skill‐building. Eligible women were participants of scheduled women's only English Conversation Circles, an informal, drop‐in group setting to practise English conversation skills held twice weekly. The agency coordinator suggested two dates when we could attend Circles and informed each of the two groups in advance about the study. On agreed upon dates, the agency coordinator introduced C (MSc, female, research associate with qualitative experience) and B (BSc, female, research coordinator) to participants at the end of the Circle session, then C and B briefly described the purpose of the study, and invited interested women to remain in the classroom for the focus group, all of whom did. C or B read an informed consent statement, and participants gave verbal consent. We planned to conduct two focus groups with a minimum total of 20 women, a common recruitment target for focus groups, who varied by age, education and country of origin.^17^ We gave women a $50 grocery chain gift card upon conclusion of the focus group. We had no prior relationship with the agency coordinator or participating women.

### 2.3 Data collection

We developed questions based on a comprehensive PCC framework that was rigorously developed by McCormack et al.^9^ with patients and clinicians, and then tailored based on interviews about PCC with diverse women who were largely Caucasian.^18^, ^19^, ^20^ The Framework of PCC for Women is organized in six domains (foster a healing relationship, exchange information, address concerns, manage uncertainty, share decisions and enable self‐care) and identifies strategies that clinicians can use to achieve each domain (File S1). We used a semi‐structured question guide (File S2) to understand whether migrant women experienced PCC according to its multidimensional components, and which domains were met or not met. At the outset, we explained that PCC is not about how doctors treat a health problem or disease; instead, PCC: ‘means how you and your doctor communicate with each other, what they do to understand your health‐care problem, and how they involve you in discussing or choosing treatment’. We first asked what they, as women, expected of their doctor for PCC. To further and more fully probe for PCC views and experiences, we then asked questions specific to each of the six PCC domains and concluded by asking what else doctors could do to improve PCC for women.

Prior to focus groups, A (PhD, female, senior scientist/professor with many years of qualitative research experience) met with C and B to review the question guide, and discuss approaches for asking questions and probing for additional information. We held three 1‐hour focus groups. C and B each led a separate focus group on 21 July 2019 because the drop‐in group that day was too large for a single focus group. After the first two simultaneous focus groups, A met with C and B to review how thoroughly each question was addressed and suggest additional probes. C and B jointly led a third focus group on 31 July 2019. Focus groups were audio‐recorded and transcribed verbatim by a professional transcriptionist.

### 2.4 Data analysis

C, B and A derived themes inductively from the data using constant comparison in iterative fashion,^21^ and used Excel to organize data. All three independently analysed one transcript to identify and code all themes, then compared and discussed themes to create a codebook of themes and exemplar quotes (level one coding). C and B independently analysing the remaining two transcripts, compared and discussed coding, then expanded or merged themes to refine the codebook (level two coding). No new themes emerged, suggesting that thematic redundancy was achieved. ARG reviewed all transcripts and the codebook to resolve flagged differences or uncertainties. We mapped themes from inductive constant comparison to PCC domains, and tabulated quotes by PCC domain and theme, described themes with exemplar quotes and noted women's experiences of each domain. We transformed positive and less positive PCC experiences reflecting behaviours of importance to women into approaches that physicians can employ to optimize PCC for migrant women.

## 3 RESULTS

### 3.1 Participants

In total, 23 women participated in three focus groups (Table 1). Median age was 56 (range 25‐78). Most were highly educated: Two high school, 10 university and 11 post‐graduate degrees. They represented 10 countries of origin: Brazil, China, Ethiopia, Honduras, India, Iran, Israel, Mexico, Romania and South Korea. Twenty‐two women had seen a doctor within the last year and one within the last 2 years.

**TABLE 1:** Participant characteristics

Characteristic	Group 1	Group 2	Group 3
Participants (n)	7	7	9
Age (median, range)	60 (56‐70)	56 (28‐78)	40 (25‐68)
Education	University 4 graduate 3	High school 2 University 2 Graduate 3	University 4 graduate 5
Country of origin	China 2 Iran 3 Israel 1 Romania 1	India 1 Iran 5 Mexico 1	Brazil 1 China 2 Ethiopia 1 Honduras 1 India 1 Iran 2 South Korea 1

### 3.2 PCC expectations

One theme pertained to variable understanding of PCC among women. A few women understood that PCC meant tailoring care to the unique needs of individuals.

Certain problem of the patient based on their needs, not based on what the doctor think it is (group 2)

However, the concept was novel for most women, who largely equated PCC with clinical care or a physical place where care was delivered, despite opening remarks in which we explained that PCC referred to interactive communication and not to treatment.

Like a centre that I can find a family physician, maybe doing also the blood test (group 1)

### 3.3 PCC experiences and recommendations by domain

File S3 includes data by PCC domain and theme. We describe themes (in italics) here by PCC domain with select quotes. Table 2 summarizes 28 themes, referring to approaches of importance to migrant women that physicians can adopt to optimize PCC across six domains.

**TABLE 2:** Framework of patient‐centred care for migrant women

PCC domains^9^	Themes	Approaches
Foster a healing relationship	Extend a greeting	Engage in friendly discussion prior to clinical discussion
	Be attentive	Make eye contact Avoid prolonged computer use
Exchange information	Listen to reason for visit	Allow patient to fully explain the health issue
	Ask questions	Prompt patient to provide additional details
	Provide detailed explanations	Fully describe symptoms and condition
	Communicate clearly	Use lay rather than medical terms Speak slowly Use patient’s first language (if possible)
	Ensure privacy	Use a private room for discussions/examination Avoid exposure to others during examinations Avoid speaking loudly to prevent sound from travelling Ask if an interpreter is wanted Provide access to women physicians or involve women nurses in discussing reason for visit
	Provide additional information	Share test results Provide written information to take away
Address concerns	Probe for concerns	Actively enquire about concerns, feelings or reactions
	Acknowledge concerns	Acknowledge, consider and respond to concerns
	Accommodate other concerns	Accommodate additional potentially‐related concerns
Manage uncertainty	Discuss benefits and risks of tests or treatment	Offer rationale for tests or treatment Describe likelihood of various outcomes
Share decisions	Provide opportunity for shared decisions	Introduce the option of taking part in decisions Assess interest in sharing decisions
	Provide information to support decisions	Describe management options Suggest factors to consider in decision making
Enable self‐care	Provide instructions	Describe procedures for tests or treatment Outline follow‐up appointments
	Offer brief counselling	Provide opportunistic counselling on prevention and screening

#### 3.3.1 Foster a healing relationship

Women described two themes: *extend a greeting* before launching into discussion of the clinical reason for the visit; and *be attentive* by facing them, not a computer, and making eye contact. Women said these behaviours showed respect and made them feel they could rely on their physician. However, several women said physicians did not practise these behaviours.

My family doctor is very busy and always when I go there, he just say hi, why are you coming here? (group 3)

#### 3.3.2 Exchange information

Women placed particular emphasis on exchanging information and articulated several themes. Women wanted physicians to *listen to the reason for the visit*, although several women said that physicians were often rushed and impatient. Given language challenges, women said they needed time to fully explain the reason for their visit.

I expect my doctor be patient and ask many question and give us enough time to explain my difficulties and problems, not to be in a rush (group 1)

Women said it was important that physicians *ask questions* to prompt them for more information, rather than waiting for them to ask questions.

Ask the patient more so that patient answers more (group 2)

Women agreed that physicians should *provide detailed explanations* about their symptoms or condition so that women fully understand the problem.

I talk with him about what am I feeling or what I have, I expect that he can be able to explain me very detailed what is happening (group 3)

Given English as a second language, many women underscored that physicians should *communicate clearly* by using plain language rather than medical jargon, and by speaking slowly to facilitate comprehension, although not all physicians did so.

I asked him to talk slowly with me but he was very busy and he didn’t accept and continued his manner (group 2)

A few women said that it was ideal when physicians communicated in their first language, but if that was not possible, they wanted physicians to *ensure privacy* and hence expressed reluctance to using a third party interpreter. Privacy concerns also meant that physicians should moderate speaking volume to prevent those in adjacent rooms from overhearing discussions.

We have the language barrier and they can bring an interpreter to help out. The privacy, maybe some woman they don’t want to let the third party to know as a woman we have problem (group 3)

And then when the doctor says something and the next room the patient can hear and this is not good, not professional and I will expect the doctor will have the privacy and don't speak loud or have the room is more privacy (group 3)

Approaches for ensuring privacy recommended by women included using a private room or space for discussions or examinations, and preventing exposure to others during physical examinations.

He should respect when he exam the woman and he should take the privacy of them, they shouldn't be exposed to the other people…some nurses or some receptionist (group 3)

Another approach to privacy was offering access to women physicians or involving women nurses in discussing the reason for the visit before seeing the physician. Notably, women uniformly referred to he/him when speaking of physicians, suggesting they may have little to no interaction with women physicians.

Some people maybe don’t like to go for a man doctor (group 2)

Maybe they have some special nurses for the woman to take a history before they meet the family doctor. Sometimes we can explain our problem with a woman, maybe more confidentially than a man (group 3)

To supplement discussion, women wanted physicians to routinely *provide additional information* such as printed test results or supplementary brochures or handouts that they could take away with them to facilitate understanding.

If I ask my doctor please give me my documents, he may give me my documents. But I think if the document give to every patient, it is more better than the patient ask them (group 2)

#### 3.3.3 Address concerns

Women said that physicians did not typically *probe for concerns*, referring to feelings or reactions about their health issue or its treatment. Moreover, several women said that it was important for physicians to *acknowledge concerns*, because they said physicians had ignored or dismissed their queries or requests for assurance.

First three months [referring to pregnancy] always have the bleeding and then make appointment to see the family doctor. And I ask maybe have a treatment, take some medicine or other things. He say no, you're just making yourself hyper and trust in god, everything will be okay (group 3)

Another theme agreed upon by many women was to *accommodate other concerns*, rather than limiting them to discussion of only one health issue.

You can say just one problem, not more. When you ask more questions, they don't like it (group 3)

#### 3.3.4 Manage uncertainty

Women uniformly said that physicians described testing or treatment options, but did not take the time to *discuss benefits and risks* of tests, treatment or other management options as a way of addressing concerns or preparing women to be involved in decision making.

Usually they don't have enough time, maybe few minutes, it's finished. For me, it's happened a lot (group 2)

#### 3.3.5 Share decisions

Some women said that physicians did *provide information to support shared decisions*, whereas other women did not routinely receive such information. One woman thought it may be ill‐advised to involve patients in decisions.

When I got some problem and then he suggests some treatment I will ask I don't know what I should do or not, and then he will never, never give me answer, he said that you decide, that's up to you is what his answer (group 3)

Many people if you give some choice for them maybe it’s not good for them because they didn't know anything about their problems and they can’t decide the best choice (group 2)

Some women said they were given the *opportunity for shared decisions*, whereas others were not.

I have seen this in movie, I haven't experience in Canada my physician wants to explain and I decide (group 1)

#### 3.3.6 Enable self‐care

Most women agreed that physicians *provided instructions* for tests or treatment, and for next steps such as follow‐up appointments.

She explain me exactly what medicine I have to take and which hour. And she ask me come next week (group 1)

A few women said that physicians should *offer brief counselling* when they attend appointments for specific health‐care issues to ensure that women adopted healthy lifestyle behaviours to prevent illness and anticipated recommended screening.

We go to physician for some specific problems, but for overall general health there should be some information provided to the woman in advance. So not specific to disease but overall general health as a woman, they should advise us (group 2)

### 3.4 Key themes and implications

Here we report overarching themes and the impact of those themes on the health‐care experience. While PCC in general was a novel concept for most women, they were able to identify PCC behaviours important to them across all domains. However, most women did not experience those behaviours, which was true across all PCC domains. The only behaviour that women uniformly experienced was *provided instructions* for tests, treatment and follow‐up appointments in the domain of enable self‐care.

While discussion largely focused on the domain of exchange information, PCC approaches across all domains were woven together by the need for physicians to allocate sufficient time for these behaviours, something that all women valued but few women experienced.

He's really nice. He take his time, he ask you how are you? How's the family? What can I do for you? Then I'll tell him my problem. And he sit down, he explain to me until I understand everything. He take his time. He's not rush you, fast, fast. He take time, he explain too everything (group 3)

Instead, women said that physicians were rushed, and ignored or dismissed their questions and concerns. Women identified three potential implications associated with these experiences that could lead to adverse health consequences. One, without sufficient time to fully explain the reason for their visit, women thought physicians might not achieve an accurate understanding of their health issue.

I'm not sure that they understand my problems because I can talk English about other things but I can't talk English in medical terms (group 2)

Two, if physicians dismissed or ignored what they said, women would avoid trying to articulate questions or concerns.

The doctor doesn't have time to hear my questions, to hear my answers. Maybe I'm not speaking good. And I would not say what I’m trying to say or what I'm feeling (group 3)

Three, women said that being rushed and dismissed led to poor patient experiences, and as a result, they may no longer seek care.

They don't have time to spend with you, they don't want to listen too much. I don't want to stay with if I have some doubts for my health issue. I try to not to see the doctor anymore because he doesn't help me (group 3)

## 4 DISCUSSION

### 4.1 Summary of findings

Through three focus groups with 23 migrant women who varied by age and country of origin, we identified 28 approaches across six PCC domains that physicians can adopt to optimize health‐care experiences for migrant women. The approaches represent behaviours that women considered important, but most women said their physicians did not employ those approaches. In particular, women emphasized the PCC domain of exchanging information, which comprised the majority of themes. However, we probed for preferences across all PCC domains, and approaches women valued across those domains pertained to physicians allocating sufficient time to discussion. Instead, women said physicians were rushed, and ignored or dismissed their questions and concerns. As a result, women said it was likely that physicians would not understand their health issue, and they would refrain from articulating health issues or details, or even avoid seeking care.

### 4.2 Comparison to other research, and implications for practice and research

These findings confirm and build upon prior research. Previous studies showed that migrants are less likely than the general population to experience high‐quality care,^2^, ^3^ in part due to language barriers among migrants, and lack of knowledge and skill among physicians about how to communicate with migrants.^4^, ^5^ Previous studies also showed that women in general, including migrant women, face gendered inequities in health‐care quality.^1^, ^6^, ^7^, ^8^ However, no earlier research provided insight on strategies to specifically optimize PCC for migrant women.^10^, ^11^, ^12^, ^14^ A scoping review identified only five studies that involved migrant women to explore PCC views and experiences.^14^ In those studies, women said that physicians dismissed their concerns and they preferred women physicians. This study confirms and expands upon those findings. It also addresses the gap in knowledge about PCC strategies by revealing approaches that physicians can adopt to optimize PCC specifically for migrant women. This study also confirms the findings of prior interviews with women whose characteristics were diverse except that they were largely Caucasian.^18^ Migrant women in this study articulated similar approaches across PCC domains, but compared with the Caucasian women in our previous study, placed greater emphasis on exchanging information and the need for privacy.

Several implications emerge from this study for policy, practice and research. Migrant women found it challenging to articulate the reason for their visit, and related concerns and questions. This may not be solely due to English as a second language because, in our prior research, women with English as their first language had similar experiences.^18^ In a survey about strategies to improve communication with immigrants, family physicians largely underscored the need for professional interpreters.^22^ In contrast, women in this study expressed reluctance to use interpreters due to a desire for privacy. Instead, interventions may be needed to prepare women for communication with physicians. There is no precedence for this.^14^ Therefore, on‐going research should identify strategies preferred by migrant women to help them gain skill in patient‐physician communication. Those findings could be used to develop and evaluate the impact of those strategies. Given that women in this study said they were reluctant to articulate health‐care issues or even to seek care, research is warranted to assess if lack of PCC among migrant women leads to adverse health consequences.

In this study, migrant women said they did not experience PCC. In prior research, we interviewed 37 physicians representing seven specialties who said they needed training n PCC and women's health.^19^ In previous research, physicians said they lacked knowledge and skills for culturally competent communication, an approach similar to PCC.^5^ Thus, physicians may benefit from education on cultural competence, leading to greater PCC for migrant women. Seeleman et al. developed a conceptual framework for teaching cultural competence and identified six approaches by which health service organizations can address diversity.^23^, ^24^ However, a Cochrane systematic review on cultural competence education for physicians (5 RCTs, 337 providers, 8400 patients) found no evidence of effect on patient satisfaction with consultations, patient scores of physician cultural competency, or clinical outcomes such as cholesterol control among patients with diabetes.^25^ Further research is needed to establish the characteristics of effective strategies for helping physicians achieve PCC. Educational interventions may be one option. Incorporating advice in clinical guidelines maybe another option, since a clinical guideline on preventive health care for migrants noted the need to provide migrant women with PCC, but did not specify how.^12^ An analysis of the content of 27 clinical guidelines found they offered little guidance on PCC for women.^26^ An underlying theme in this study was insufficient time for thorough communication. Thus, additional strategies may be needed at the system‐level to promote models of care that include longer physician appointments or multidisciplinary appointments in which nurses assume some aspects of consultation and care, as was suggested by women in this study.

Migrant women in this study said they would feel more comfortable speaking with and being examined by a woman physician. Most referred to physicians as he or him, suggesting they largely had experience with men physicians. Previous research also found that immigrant women favour a woman physician.^27^ A 2015 report noted that women comprised 55% of family medicine trainees in the United States,^26^ but women are underrepresented in rural areas and other specialties.^28^, ^29^ The issue is controversial due to concerns about shortfalls in medical human resources. A systematic review of 34 studies published from 1991 to 2013 found that women primary care physicians self‐reported fewer hours of work, patient encounters, and services delivered compared with men physicians, but longer visit time and discussion of multiple problems with individual patients, qualities desired by migrant women in this study.^30^, ^31^ Future research is needed to ascertain how to support and retain women physicians and how to enhance access to women physicians for migrant women.

### 4.3 Study strengths and limitations

The study features multiple strengths. We employed rigorous research methods and complied with qualitative research reporting criteria.^15^, ^16^, ^17^ We included migrant women of diverse age and country of origin who expressed strong agreement, which supports broad relevance of the findings. We mapped findings to a PCC framework as a means of thoroughly exploring elements of the health‐care experience important to migrant women.^9^, ^18^, ^19^, ^20^ In so doing, we further validated the Framework of PCC for Women and revealed key elements of PCC desired by migrant women. As a practical output, we generated 28 approaches that physicians can adopt to optimize PCC for migrant women. Some limitations must also be mentioned. Participants were volunteers, and thus possibly motivated by a particular interest in PCC or a desire to share poor health‐care experiences. In our attempt to explain PCC, we may have failed to convey its full meaning, leading women to focus on the exchanging information domain. However, we did prompt migrant women to discuss each of the PCC domains, and these findings confirm and elaborate on prior similar research that involved largely Caucasian women, underscoring the importance of two‐way communication.^18^ Participating migrant women were highly educated, so their views and experiences may not reflect those of migrant women with less education. The women all lived in <city, country>, so findings may not be transferrable to migrant women elsewhere in <country>, or in other countries with different health‐care systems. While PCC may also be a relevant issue for migrant men, this study specifically focused on the aspects of PCC that migrant women value.

## 5 CONCLUSIONS

Twenty‐three migrant women aged 25‐78 from 10 origin countries who participated in three focus groups identified 28 approaches across six domains that they viewed as essential elements of PCC, but largely did not experience in interactions with physicians. Instead, they said physicians rushed through discussions, and ignored or dismissed their concerns and questions. In particular, women emphasized the domain of exchanging information, which included 13 (46.4%) of 28 total approaches across the following six themes representing physician behaviour: listen to reason for visit, ask questions, provided detailed explanations, communicate clearly, ensure privacy and provide additional information. Women identified potential adverse consequences as a result of poor PCC: physicians may not fully understand their health issue, and they may refrain from articulating important details or even avoid seeking care. By revealing specific physician behaviours to optimize PCC for migrant women, this research addresses a void in prior research. Future research is needed to develop and evaluate the impact of women, physician and system‐level strategies to support PCC for migrant women.

## CONFLICT OF INTEREST

The authors declare no conflicts of interest.

## Supporting information

File S1

File S2

File S3

## Data Availability

Data are available in article Supplementary material.
