# Supplementary material for: Physician behaviours that optimize patient‐centred care: Focus groups with migrant women
Source: Health Expect. 2020 Jul 24;23(5):1280–8. doi: 10.1111/hex.13110 (PMC7696129; doi:10.1111/hex.13110)
Supplement: Supplementary file 1 — File S1 [file HEX-23-1280-s001.docx]

Additional File 1. Framework of patient-centred care for women [18-20]

| PCC domains [9] | Themes | Approaches |
| --- | --- | --- |
| Foster a healing relationship | Establish rapport | - Engage in friendly discussion prior to clinical discussion |
|  | Assume a non-judgmental attitude | - Maintain a neutral disposition - Speak in a respectful manner |
| Exchange information | Learn about context | - Allocate time to explore patient/caregiver context - Learn about lifestyle, social circumstances, personal goals, etc. |
|  | Allow time for discussion | - Avoid conveying a sense of rush to see the next person - Ask if there are remaining questions |
|  | Demonstrate active listening | - Sit facing the person and make eye contact - Record notes only after the person finishes speaking |
| Address concerns | Elicit emotions or concerns | - Take a holistic approach to care - Explicitly ask about feelings |
|  | Validate emotions or concerns | - Acknowledge hearing and understanding concerns - Reassure that it was appropriate to mention those feelings - Note that such feelings are normal or common |
| Manage uncertainty | Identify uncertainties or risks | - Explicitly note uncertainties about prognosis, and the risks and benefits of treatment options - Acknowledge the possibility of adverse outcomes |
|  | Discuss uncertainties or risks | - Describe the likelihood and nature of adverse outcomes - Answer questions about uncertainties or risks |
| Share decisions | Identify treatment or management options | - Note treatment or management options - Describe which are suitable or recommended, and why |
|  | Offer participatory decision-making | - Explore opinions or preferences about options - Invite them to make the final decision |
| Enable self-care | Describe the follow-up process | - Explain follow-up appointments (who to see, when) - Specify who to contact and how if concerns or issues arise |
|  | Offer self-care advice and instruction | - Provide verbal guidance on self-care and self-monitoring - Provide or offer referral to educational material or seminars |
