# Supplementary material for: Physician behaviours that optimize patient‐centred care: Focus groups with migrant women
Source: Health Expect. 2020 Jul 24;23(5):1280–8. doi: 10.1111/hex.13110 (PMC7696129; doi:10.1111/hex.13110)
Supplement: Supplementary file 2 — File S2 [file HEX-23-1280-s002.docx]

Additional File 2. Focus group question guide

Opening Remarks

Thank you for agreeing to speak with me. The purpose is to understand how to improve patient-centred care (PCC) for women. We are not asking about what they do to treat a health problem or disease. PCC means how you and your doctor communicate with each other, what they do to understand your health care problem, and how they involved you in discussing or choosing treatment. I will ask you several questions about communication with your doctor to find out what was good or what could be better for you as a woman. To answer the questions, try to think about the most recent appointment where you had to see the doctor for a health issue you had. Please try to provide details, not just “yes” or “no”; try to explain to us how you think or feel. Everything you tell us is confidential. That means we will never reveal your names. Please speak loudly so that your voice can be picked up by the recorder. Do you have any questions?

General PCC question

As a woman, what do you expect from your doctor for patient-centred care?

Prompts: what do they do to help you discuss or learn about a healthcare problem or how to treat it, ask questions, understand what to do about the problem

Domain-specific questions [for each; if not, what should they have done?]

What did your doctor do to get to know you before talking about your health problem?

Prompts: What did they do or say to make you feel welcome and comfortable? How did they get to know you?

How did your doctor discuss the health problem with you?

Prompts: How did they ask you to describe the problem or what you think might be the cause? Did you have enough time to do that? Did they invite you to ask questions? How did they provide information to you about the problem and treatment options?

How did your doctor assess if you were worried or anxious about your health problem?

Prompts: emotions/concerns? Did they ask you if you were worried, or to describe your worries? Did they listen? Did they offer suggestions or information to reduce worries?

How did your doctor explain how well the treatment might work?

Prompts: benefits/risks/harms, did they provide any information about improvement or side effects in words or numbers?

How did your doctor involve you in making decisions about your treatment?

Prompts: Did they ask for your opinion about the treatment or what you might prefer?

How did your doctor prepare you to take the treatment or take care of yourself?

Prompts: Did they explain next steps? If and when they need to see you again? What you should do to take care of your health? Provide you with information, or tell you where to get more information or help?

PCC recommendations

What should doctors do to improve patient-centred care for women?

Prompts: What could your doctor do better? What would help you to communicate with your doctor?

Conclusion

That concludes our discussion. Thank you very much for speaking with us. Do you have any questions or anything else you would like to add?
