# Supplementary material for: Physician behaviours that optimize patient‐centred care: Focus groups with migrant women
Source: Health Expect. 2020 Jul 24;23(5):1280–8. doi: 10.1111/hex.13110 (PMC7696129; doi:10.1111/hex.13110)
Supplement: Supplementary file 3 — File S3 [file HEX-23-1280-s003.docx]

Additional File 3. Themes and quotes

| Domain | Theme | Quotes |
| --- | --- | --- |
| Foster a healing relationship | Extend a greeting | Usually they respect us at first. They say how are you, I’d say, oh I’m okay, and after that we talk about our problems (group 2)  My family doctor is very busy and always when I go there; just say hi, why are you coming here? (group 3)  He take his time when you go inside, he ask you how are you? How’s the family? And everything. And he see his file, then he check, okay …what can I do for you. Then I’ll tell him my problem (group 3) |
|  | Be attentive | Contact eyes is very good, I can rely to my doctor when he have eye contact with me and I feel that he pays attention to me (group 2)  When you arrive there, they look at the computer and they look at our electronic profile, and after that they start to ask us about our problems (group 3)  When I last time went to doctor, see my baby’s vaccination, they see the laptop. Always look like this all the time [mimicked looking at computer] (group 3) |
| Exchange information | Listen to reason for visit | I expect my doctor be patient and ask many question and give us enough time to explain my difficulties and problems, not to be in a rush (group 1)  Yah, my doctor is friendly too and she gave us 20-minutes; 20-minutes for every patient and talked to her, it’s easy for me because she listen to my problem (group 1)  My doctor ask about my problem and he’s patient and I describe him my situation and problem (group 1)  I’m not sure that they understand my problems because I can talk English about other things but I can’t talk English in medical terms (group 2)  I told the family doctor my symptom, but always the doctor is very busy. So I can’t say my symptom clearly. So I have to speak very shortly and then he just prescribe some medicine (group 3)  To spend the time and hear very well, and after that explain about the problem clearly (group 3) |
|  | Ask questions | I want my doctor not only to solve the problem that I came for. I want him to ask me as many questions as they can in order to find if I have other problem (group 1)  Actually my family doctor is very friendly, after she have…listen to our problem if I have patient. And she can give me all of the information about my problem and I’m happy with her (group 1)  Ask the patient more so that patient answers more (group 2)  I think it’s very important in patient centered care to ask question and listen to the patient as well (group 2)  I have a very good family doctor. He takes his time and explain everything and ask a lot of questions. It’s very good (group 3) |
|  | Provide detailed explanations | I talk with him about what am I feeling or what I have, I expect that he can be able to explain me very detailed what is happening (group 3)  I have something bad, I don’t feel well, so I expect the doctor will explain to me, clearly or more in detail what’s going on with my symptoms (group 3)  I expect that my doctor or my family doctor will be able to explain me all the things related to my situation you know? (group 3) |
|  | Communicate clearly | I asked him to speak slowly because I can’t understand him when he talks so fast and I wanted to understand (group 2)  She explained very patient with me because my English is not good so please talking slowly (group 2)  I asked him to talk slowly with me but he was very busy and he didn’t accept and continued his manner (group 2)  It is very, very important because if the patient cannot understand what the doctor says, it is not good (group 2)  He should explain the issue to the patient as a normal language, not as professional language (group 3) |
|  | Communicate in patient’s first language | He will be awesome if he can explain me that in my language (group 3)  I want they speak my language because maybe they have a lot of terminology that I don’t understand if English is not my first language (group 3) |
|  | Ensure privacy | I think doctors must respect to their patients. For example, speak with them privately (group 2)  Some people maybe don’t like to go for a man doctor (group 2)  And then when the doctor says something and the next room the patient can hear and this is not good, not professional and I will expect the doctor will have the privacy and don’t speak loud or have the room is more privacy (group 3)  We have the language barrier and they can bring an interpreter to help out. The privacy, maybe some woman they don’t want to let the third party to know as a woman we have problem (group 3)  Maybe they have some special nurses for the woman to take a history before they meet the family doctor and it’s better than they are woman, the special nurse I mean, because you know sometimes we can explain our problem with a woman, maybe more confidentially than a man (group 3)  It’s good that you have some privacy in the clinic when doctor want to exam you, bring you to another room that is special for the woman (group 3)  When a woman talk to a doctor especially when he is a male, he should respect when he exam, he or she, doesn’t matter, exam the woman and he should take the privacy of them, they shouldn’t be exposed to the other people. It doesn’t matter who the people are, some nurses or some receptionist, it doesn’t matter (group 3) |
|  | Provide additional information | I want my family doctor to tell me about many information. Doctor in Canada they don’t give you any information (group 1)  I think every document is give to every patient, for example, of the laboratory exam. In Canada, if I ask my doctor please give me my documents, he may give me my documents. But I think if the document give to every patient, it is more better than the patient ask them (group 2)  sometimes the doctor only explain and they don’t give you nothing, like a flyer or brochures or something that can be helpful for you to get the right information (group 3) |
| Address concerns | Acknowledge concerns | The last time I went there I had shoulder pain, he just looked at my profile and said it doesn’t matter and it is not a big problem, maybe you have a bad sleep and he recommended me a cream. And I said to him that I think it is not muscular but he didn’t pay attention (group 3)  First three months [referring to pregnancy] always have the bleeding and then make appointment to see the family doctor. And I ask maybe have a treatment, take some medicine or other things. He say no, you’re just making yourself hyper and trust in god, everything will be okay (group 3)  I really want to have people to take care of me, to follow-up with my, like testing or everything. Family doctor, he very, very nice but he just always say you are young, don’t worry. Yah, before I asked him to transfer me to the special doctor and he say you don’t need that, you’re very healthy, you can live long, long, long time. And I say but I would like to know the result is not very serious (group 3) |
|  | Probe for concerns | I don’t think is a talking about my emotional feeling, about my concern (group 1)  So it’s not directly that they asking me, so how you feel about it or what is your worry about your situation (group 1)  Usually physicians care only about the physical symptoms and not ask how it’s affecting you, is it worrying for you (group 2)  My family doctor never ask about that (group 3) |
|  | Accommodate other concerns | He wants you to finish your appointment with him, like after 10-minutes because he had other people to see (group 1)  Not enough time to ask something more than the original problem we have for something out of these questions (group 2)  Actually they [primary healthcare provider] say just one complain, not any other complain… (group 2)  I expect the doctor have time to listen to me and listen to my symptoms. Sometimes when I go to a walk-in clinic they tell you just to pick up one symptom and they are always now rush (group 3)  One bad thing is for the family doctor, if you have a few things together and to ask. And then he will stop you because he just allow you to say one issue and then if more than that, he will not listen (group 3)  You can say just one problem, not more. When you ask more questions, they don’t like it (group 3)  He say, okay just focus on one problem just for two minutes. And say okay if you don’t have any question, I have to go to the other patient (group 3)  Just two question, maximum three and then more than that, stop you (group 3) |
| Manage uncertainty | Discuss benefits and risks of tests or treatment | Mostly they explain about the treatment (group 1)  Usually they don’t have enough time, maybe few minutes, it’s finished. For me, it’s happened a lot (group 2)  Didn’t explain for me, just gave me the paper and he say you have to read it, that’s it (group 3)  He said if you want to do the ultrasound or do the x-ray and then I can give you the paper to do it. And he didn’t really take the time to explain to me what is the benefit (group 3) |
| Share decisions | Provide opportunity for shared decisions | No  I have seen this in movie, I haven’t experience in Canada my physician wants to explain and I decide (group 1)  So I follow the doctor”s opinion (group 3)  Yes  They [primary healthcare providers] recommend things but they let you decide. So he explain about other, other exam that I can take instead of the colonoscopy but he leaves me to decide if I want to do or not (group 1)  He [primary healthcare provider] gave me the chance to choose between his decisions (group 2)  Sometimes my doctor is so good, he writes down in a paper so we can decide together what’s the better option (group 3)  Maybe not a good idea  Many people if you give some choice for them maybe it’s not good for them because they didn’t know anything about their problems and they can’t decide the best choice (group 2) |
|  | Provide information to support decisions | They explain every option that the patients can choose one by own (group 2)  When I got some problem and then he suggests some treatment I will ask I don’t know what I should do or not, and then he will never, never give me answer, he said that you decide, that’s up to you is what his answer (group 3) |
| Enable self-care | Provide instructions | She explain me exactly what medicine I have to take and which hour. And she ask me come next week (group 1)  So they explain what you need to do, which diet takes, prefer to take and they said, come in three months to another check-up. (group 1)  She explained me what has happened, what’s the medicine and how do I have to take it, what will be happening after that. And she told me if you still feel bad you have to come again and we have to do the exams again (group 3)  And I think she was very clear explaining about the treatment and what do I have to do after that (group 3) |
|  | Offer brief counseling | We go to physician for some specific problems, but for overall general health there should be some information provided to the woman in advance. If someone is going through menopause, then what will happen to you. Also, about deficiencies, like iron deficiencies, we have all the sufficient nutrients or not about our diet. So not specific to disease but overall general health as a woman, they should advise us (group 2)  You can see your document, you need to do some test and every year or maybe three or four month, because we don’t know if the doctor don’t tell us (group 3) |
